# Intestinal microsporidiosis in Strasbourg from 2014 to 2016: emergence of an *Enterocytozoon bieneusi* genotype of Asian origin

**DOI:** 10.1038/s41426-018-0099-9

**Published:** 2018-06-06

**Authors:** Valentin Greigert, Alexander W. Pfaff, Ahmed Abou-Bacar, Ermanno Candolfi, Julie Brunet

**Affiliations:** 1 Infectious Diseases Department, Hôpitaux Civils de Colmar, 39 Avenue de la Liberté, Colmar, France; 2 Institute of Parasitology and Tropical Diseases, EA 7292, University of Strasbourg, 3 rue Koeberlé, Strasbourg, France; 3 Department of Parasitology and Mycology, University Hospital of Strasbourg, 3 rue Koeberlé, Strasbourg, France

## Abstract

Microsporidia cause opportunistic infections in highly immunodeficient individuals. Few studies on the epidemiology of these infections have been conducted in France. Between 2014 and 2016, we undertook a study to estimate the prevalence and circulating genotypes of this fungus-related micro-organism among the population of Strasbourg University Hospital. Samples were collected from hospitalized patients and analyzed using microscopy and molecular assays. Strains from positive subjects were sequenced for genotyping. Only 7/661 patients (1.1%) were positive for microsporidia, and the only species identified was *Enterocytozoon bieneusi*. Two patients presented immunodeficiency linked to AIDS, and five transplant recipients presented immunodeficiency linked to immunosuppressive therapies. Only five patients received specific antimicrosporidial treatment, but clinical outcomes were good in all cases. We identified four genotypes: A and D in patients with AIDS, and C and S9 in transplant recipients. To date, genotype S9 has been described only once. This genotype is similar to those found in farm animals in China. Because some of these animals have been introduced to Central Europe, we postulate that this genotype might be of Asian origin. Thus, genotyping microsporidial strains may be of epidemiological and clinical interest to identify potential outbreak sources depending on the infecting strains.

## Introduction

Microsporidia are a group of unicellular fungi living as obligate intracellular parasites^1^. The first microsporidial species to be described, *Nosema bombycis*, was identified by Carl Wilhelm von Nägeli in 1857^2^ as the causative agent of a silkworm disease. Since then, approximately 1000–1500 microsporidial species have been described^1^. Over the past 150 years, microsporidia classification has been widely discussed, based on morphological, biochemical, and genetic characteristics. Although Nägeli^2^ initially placed *Nosema bombycis* in the fungal group, *Schizomycetes*, microsporidia were considered protozoans for more than a century^1^. Recently, phylogenetic analyses of five gene sequences (mitochondrial HSP70, TATA-box protein, RNA polymerase II, and α-tubulin and β-tubulin) have supported classifying microsporidia within the fungi or as a sister-group with a common ancestor^1^.

Until the 1970s, microsporidial species were rarely recognized as causes of human pathology^1^. With the HIV epidemic emerging in the first half of the 1980s, microsporidia, which cause opportunistic infections in highly immunodeficient individuals, became more frequent and visible, concomitant with physicians’ increasing knowledge of this condition^1^. Thus, diverse symptoms were associated with microsporidial infections, most commonly digestive disorders, but many other unusual symptoms as well^1^. Four species primarily cause human infections: *Enterocytozoon bieneusi, Encephalitozoon intestinalis*, *Encephalitozoon hellem*, and *Encephalitozoon cuniculi*.

Few studies on the epidemiology of these infections have been conducted in France, and most of these studies were on microsporidial infections in HIV-infected patients. However, current patients undergoing immunosuppressive therapies, such as transplant recipients, account for many of the immunocompromised patients, and microsporidial infections have been described under these conditions^3^. In this retrospective study, we evaluated the frequency of these infections in Eastern France by analyzing cases diagnosed between January 2014 and December 2016 at the Strasbourg University Hospital. We were interested in epidemiological, clinical, and microbiological features, including genotyping, to determine the current burden and characteristics of these infections in Eastern France.

## Results

### Clinical features

Between January 2014 and December 2016, 661 samples were sent to the laboratory of the University Hospital of Strasbourg for microsporidia testing. Of these, 613 (92.7%) came from immunocompromised patients, including most organ transplant recipients (61.1%) (Table 1). Samples were taken from 281 females (42.5%) and 380 males, with an average age of 52.27 years (range 16 days–92 years), and 57 samples (8.6%) were from patients under 18-years old. Only seven patients (1.1%) were positive for microsporidia, and the only species detected was *E. bieneusi*. The positive individuals’ ages ranged from 7 to 63 years, and there were 5 males and 2 females (Table 2).

**Table 1:** Underlying conditions of patients tested for intestinal microsporidiosis

Underlying immunodeficiency	613 (92.7%)
Solid organ transplant	404 (61.1%)
Solid cancer	49 (7.4%)
Malignant hemopathy	39 (5.9%)
HIV infection	39 (5.9%)
T4 cells <200/mm^3^	15 (2.3%)
T4 cells >200/mm^3^	24 (3.6%)
Hematopoietic stem cell transplant	33 (5.0%)
Autoinflammatory or autoimmune disease	29 (4.4%)
Kidney failure	5 (0.8%)
Corticosteroid treatment	4 (0.6%)
Other infection	4 (0.6%)
Malnutrition	3 (0.4%)
Humoral immune deficiency	3 (0.4%)
Alcoholic liver cirrhosis	1 (0.2%)
No immunodeficiency	42 (6.4%)
No information	7 (1.1%)
Total	662

The total is 662 because one subject infected with HIV also received a hematopoietic stem cell transplant

**Table 2:** Patients positive for *E. bieneusi*

Subject	Age	Sex	Immuno-deficiency	Symptoms	Genotype	Diagnostic method	Specific treatment	Clinical outcome	Biological outcome
1	28	F	AIDS	Acute diarrhea	D	PCR microscopy	ALBENDAZOLE 400 mg b.i.d., 3 days	Success	No control
2	55	M	Immune-suppressive therapy	Acute diarrhea	C	PCR	NITAZOXANIDE 500 mg b.i.d., 14 days + ALBENDAZOLE 400 mg b.i.d., 10 days	Success	Failure
3	58	F	Immune-suppressive therapy	Chronic diarrhea	S9	PCR	None	Success	No control
4	7	M	Immune-suppressive therapy	Chronic diarrhea	S9	PCR	Unknown	Success	No control
5	43	M	AIDS	“Yellow” stool	A	PCR	ALBENDAZOLE 400 mg b.i.d., 21 days	Success	Failure
6	57	M	Immune-suppressive therapy	Acute diarrhea	C	PCR	NITAZOXANIDE 500 mg b.i.d., 15 days	Success	No control
7	63	M	Immune-suppressive therapy	Acute diarrhea	C	PCR	None	Success	Success

*M* male, *F* female, *AIDS* acquired immune deficiency syndrome

Clinical outcomes: “success” refers to the resolution of all digestive symptoms. Biological outcome: “success” and “failure” refer to the absence or presence, respectively, of microsporidia species from the *Enterocytozoon* or *Encephalitozoon* genera in patient stool samples

All patients were immunodeficient; two patients were linked to HIV infection with AIDS (C3 stage), and the other five were linked to immunosuppressive therapies. The two patients with HIV had very low CD4 counts (38 and 61/mm^3^). For patients undergoing immunosuppressive therapies, these treatments were administered to prevent organ rejection after transplantation (four kidneys, one heart), and all five patients were receiving at least a 2-drug regimen, including mycophenolate, tacrolimus, everolimus, ciclosporin, and prednisone (Table 2).

All seven patients presented digestive symptoms with acute diarrhea (three patients), chronic diarrhea (three patients), or “yellow” stool (one patient). Six patients presented with biological inflammatory syndrome with moderately elevated C-reactive protein (CRP) ranging from 24.5 mg/l to 109 mg/l. All positive patients presented comorbidities (tuberculosis, hepatitis B and C, pneumonia, pericarditis, pneumocystosis, macrophage activation syndrome, cryptococcosis, and transplant rejection).

Five patients received specific antimicrosporidial treatment with four regimens (Table 2). We could not access treatment information for one patient because of incomplete medical records, and two patients received no specific treatment for the *E. bieneusi* infection; however, these three subjects were treated for other infections: one with azithromycin, one with azithromycin and trimethoprim-sulfamethoxazole, and the last with spiramycin, cefotaxime, vancomycin, and valganciclovir.

The clinical outcomes were good with digestive symptoms being resolved in all cases. Three patients received a second parasitological control examination 2–6 weeks after diagnosis. Two remained positive at this examination.

### Microbiological features

**Fig. 1:**
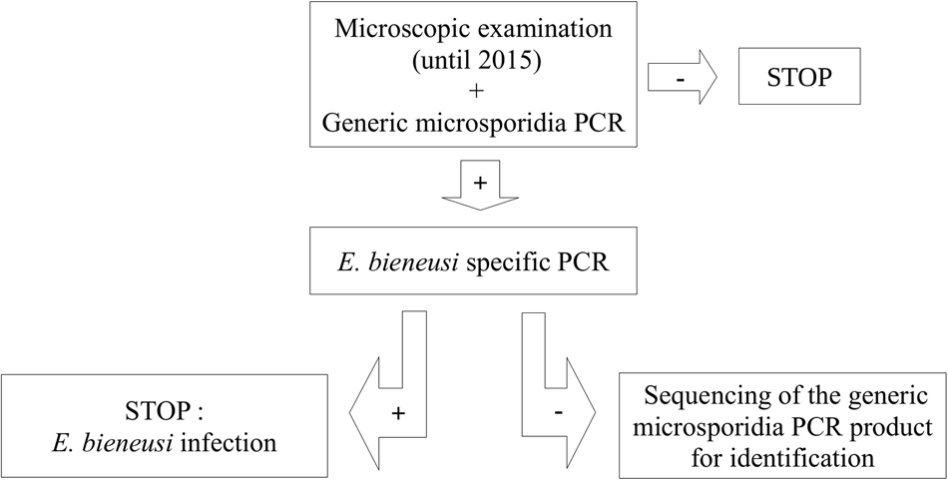
**Diagnostic procedure for intestinal microsporidiosis**

Microsporidia was diagnosed using two polymerase chain reactions (PCRs), the first to detect microsporidial species from the *Enterocytozoon* and *Encephalitozoon* genera and the second specific for *E. bieneusi* only^4^ (Fig. 1). All subjects positive for microsporidia were infected with *E. bieneusi*. Of the 661 samples examined, 46 (6.9%) were positive for other parasites. Only one (subject 5) was coinfected with *E. bieneusi*, *Blastocystis* spp., and *Chilomastix mesnili*. Eight patients were coinfected with 2–3 parasites but not *E. bieneusi*. All other patients positive for parasites were mono-infected (detailed in Table 3).

**Table 3:** All parasite results from 661 patient stool samples

	Number (/661)	Prevalence (%)
Parasites	46	6.9
Protozoa	37	5.6
* Blastocystis* spp.	17	2.6
Amebas	7	1.1
* Entamoeba coli*	1	0.15
* Entamoeba dispar*	2	0.3
* Entamoeba hartmanni*	1	0.15
* Endolimax nana*	2	0.3
Undefined ameba	1	0.15
Flagellates	7	1.1
* Dientamoeba fragilis*	1	0.15
* Chilomastix mesnili*	2	0.3
* Giardia intestinalis*	4	0.6
* Cystoisospora belli*	1	0.15
* Cryptosporidium* spp.	12	1.8
Helminths	4	0.6
Nematodes	3	0.45
Ancylostomidae	1	0.15
* Enterobius vermicularis*	1	0.15
* Trichuris trichiura*	1	0.15
Tenia	1	0.15
* Diphyllobothrium latum*	1	0.15
Fungi	7	1.1
* Enterocytozoon bieneusi*	7	1.1

**Fig. 2:**
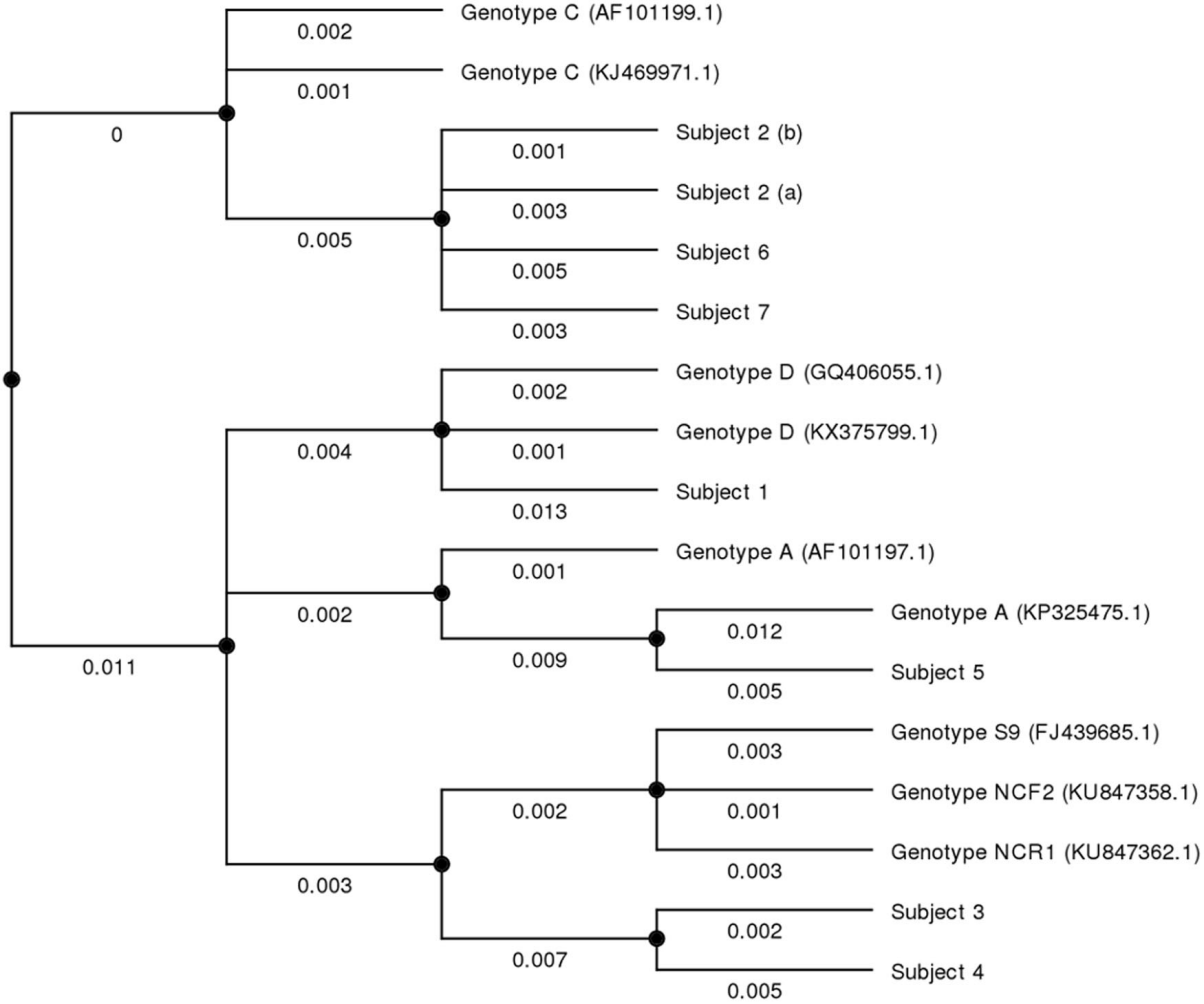
Bayesian inference phylogeny of small-subunit rRNA gene internal transcribed spacer from the strains isolated from patients and similar strains estimated using the program MrBayes with the general time reversible (GTR) substitution model. Numbers under the lines show relative distances between the strains. Study patients are marked 1–7. Reference sequences were those used by Santín et al. for *E. bieneusi* nomenclature consensus^26^ and are marked with their *GenBank* accession number

To genotype the isolated strains, another PCR was performed using primers targeting internal transcribed spacers (ITS) between small-subunit rRNA and large-subunit rRNA genes^5^. This procedure allowed comparison with published sequences in the *GenBank* database. All sequences were genetically similar, but four genotypes were identified (Fig. 2). One HIV-positive patient was infected with *E. bieneusi* genotype D, the other with genotype A. Among the five patients receiving immunosuppressive therapies, three were infected with *E. bieneusi* genotype C (all were kidney-transplant recipients) and two with genotype S9 (one kidney transplant and one heart transplant recipient). Subject 2 tested positive twice for genotype C. Subject 5 tested positive twice for *E. bieneusi*, with genotype A for the first sample; however, the volume of the second sample was insufficient and could not be genotyped.

## Discussion

We report seven cases of intestinal microsporidiosis due to *E. bieneusi* in immunodeficient patients. Most patients were transplant recipients with marked immunodeficiency mediated by anti-organ-rejection therapies. With the development of HIV protease inhibitors in 1995^6^ and the use of combined antiretroviral therapy (cART)^7^, subjects with HIV have become less susceptible to microsporidial disease^8–12^. More recently, powerful immunosuppressive therapeutics were used to prevent organ rejection after transplantation^13^, putting transplant recipients at risk of infection^14^. In addition, HIV-positive patients with very low CD4+ cell counts remain at risk. All patients who were screened for microsporidia presented symptoms attributable to *E. bieneusi* infection, which is known to cause a relatively localized disease, with a tropism for the gastrointestinal tract^1^. Diagnoses were performed using microscopy (until 2015) and PCR; however, this search was positive only 10 times for 7 patients over 3 years, with 661 tests performed. Thus, microsporidial disease seems to be an uncommon diagnosis at our hospital.

Only four of the seven diagnosed patients were specifically treated for *E. bieneusi* infection, with four drug regimens using nitazoxanide and/or albendazole. Two patients remained untreated and we found no information on the last patient. Outcomes were good in all cases. Currently, France has no official treatment recommendation for intestinal microsporidiosis or *E. bieneusi* infection; the most commonly used drugs are nitazoxanide^15, 16^ and albendazole^17, 18^. However, several successful treatments have been reported using fumagillin for intestinal microsporidiosis due to *E. bieneusi* in transplant recipients^19–23^. Furthermore, in most previously reported cases, immunosuppressive therapies were tapered or discontinued at the same time treatment was introduced^20^. Similarly, in our study, cART was initiated in HIV-infected patients, and immunosuppressive therapies were tapered in transplant recipients. Since introducing cART, many positive outcomes have been reported for intestinal microsporidiosis after immune restoration, with no other specific treatment^8–12^. However, a recent publication showed that intestinal microsporidiosis due to *E. bieneusi* in a stem cell transplant recipient was successfully treated with fumagillin and avoided modifications of immunosuppression^23^. In any case, patient immunity seems to be a key element in curing this infection. Finally, given the low prevalence of *E. bieneusi* infections in patients with diarrhea in our study, directly implicating this microorganism in intestinal symptom development in these patients remains uncertain. All samples analyzed were from patients experiencing diarrhea, but only 7 (1.1%) were positive for *E. bieneusi*. These patients may have only been carriers of this microorganism. These elements raise the question of the value of diagnosing intestinal microsporidiosis and using specific treatments.

We identified *E. bieneusi* strains that were genetically similar to four described genotypes: A, C, D, and S9. The most predominant genotype was C, found in three patients, all of whom were kidney transplant recipients. Genotype C is the most common genotype in kidney transplant patients, with a high specificity for this immune background^5, 24^, as well as for liver transplant recipients^25^. We described one patient infected with genotype A and one with genotype D, both being HIV-infected with AIDS. These genotypes are frequently observed in HIV-infected patients^5^; however, we did not isolate the most commonly described genotype in this HIV setting, genotype B^5, 26, 27^. The fourth genotype was S9, carried by two patients undergoing immunosuppressive therapies, one being a heart transplant recipient, the other a kidney transplant recipient. To our knowledge, this genotype has only been described once, in a patient with ulcerative colitis in the Netherlands^5^. This genotype is very similar to two other recently described genotypes, NCR1 and NCF2, found in raccoon dogs (*Nyctereutes procyonoides*) farmed in Northern China^28^. Other highly similar strains to these three genotypes have mostly been found in animals in China^29–31^ and recently in wild animals, especially wild boars (*Sus scrofa*) and raccoons (*Procyon lotor*) introduced in Central Europe^32, 33^. With Strasbourg being on the German border, these strains are likely to circulate in our region, possibly after having been introduced from Asia. It would be interesting to genotype more strains isolated from patients from the Rhine valley and Central Europe to assess whether these strains occur frequently in those areas.

Furthermore, outbreaks related to environmental contamination can occur in immunodeficient and immunocompetent individuals^34^. Although infections are believed to occur via ingestion^1, 35^, *E. bieneusi* can infect other mammals, birds, insects, and arthropods, making the contamination source difficult to ascertain, with a potential for zoonotic and arthropod-borne transmission^18^. Thus, this makes it difficult to recommend preventive behaviors to patients potentially at risk of microsporidial infection, and strain genotyping should be routinely performed to identify environmental sources that may cause outbreaks.

In conclusion, given its epidemiological characteristics linked to immunocompromised hosts, intestinal microsporidiosis due to *E. bieneusi* is an uncommon but potentially emerging disease with increased use of immunosuppressive treatments. Our study shows that, in our region, transplant recipients are the main population at risk of infection. The absence of treatment recommendations leaves physicians with the choice of a drug regimen that does not appear to affect the usually favorable course of infection, and patient immunity might be a key element in triggering and curing this infection. In this context, genotyping strains isolated from patients may help identify environmental sources that could potentially initiate outbreaks. Finally, we identified strains similar to uncommon genotypes that may have been introduced through animals imported from Asia. This would show the importance of human activities in spreading human and animal pathogens.

## Materials and methods

### Sample collection

All samples were collected from patients at the University Hospital of Strasbourg per standard procedures over a 3-year period (January 2014 to December 2016). Subjects were included when their clinician specifically requested testing for microsporidia.

### Microscopic examination

A first microscopic examination was performed without staining shortly after stool collection. Samples were then examined after merthiolate-iodine-formaldehyde staining and concentration (Faust-Ingalls method). From January to December 2014, all samples were also stained with Weber’s modified trichrome for microsporidial spore staining before microscopic examination by a trained parasitologist. This step was discontinued in 2015 due to a change in legislation prohibiting use of the toxic reagents necessary for Weber’s staining and because no superiority in sensitivity or specificity has been found compared with PCR^36^.

### Molecular assays

An aliquot of each stool sample was stored at −20 °C until molecular diagnosis. DNA was extracted using the DNA Stool minikit (Qiagen, the Netherlands) per the manufacturer’s recommendations, without prior mechanical disruption. All samples were submitted to a first PCR using a commercial “microsporidia generic” real-time PCR kit (Bio-Evolution, France) to detect all species belonging to the *Enterocytozoon* and *Encephalitozoon* genera and amplifying a sequence of the 18S rRNA gene (Se = 88%, Sp = 83%) per the manufacturer’s recommendations. Positive samples were submitted to a second PCR specific for *E. bieneusi*. We used the primers Eb.gc (5′-TCAGTTTTGGGTGTGGTATCGG-3′) and Eb.gt (5′-GCTACCCATACACACATCATTC-3′), which amplify a 210-bp DNA fragment in ITS sequence of *E. bieneusi*, as previously described^4^. All PCR reactions were performed using GoTaq (Promega, USA) polymerase. To genotype the different strains found in the samples, we performed a third PCR on the ITS sequence (*GenBank* accession no. AF101200). The forward primer, Eb-80F (5′-GTTGGAGAACCAGCTGAAGGT-3′), and reverse primer, Eb-375R (5′-ATACACCTCTTGATGGCACCCT-3′), amplified a 296-bp fragment, as previously described^5^. The latter PCR product was sent for sequencing (GATC Biotech AG, Germany).

Sequences obtained were compared with the *GenBank* database (www.ncbi.nlm.nih.gov/genbank/) and aligned to draw a phylogenic tree comparing the sequences. This tree was designed with the MrBayes 3.2 phylogenetic program^37^ performed through the open-source bioinformatics software, UGENE (Unipro, Russia)^38^.

### Data availability

All data supporting these findings are available without restriction upon request to the authors.
